# Two Improvements of an Operational Two-Layer Model for Terrestrial Surface Heat Flux Retrieval

**DOI:** 10.3390/s8106165

**Published:** 2008-10-01

**Authors:** Renhua Zhang, Jing Tian, Hongbo Su, Xiaomin Sun, Shaohui Chen, Jun Xia

**Affiliations:** 1 Key Laboratory of Water Cycle and Related Land Surface Processes, Institute of Geographical Sciences and Natural Resources Research, Chinese Academy of Sciences, Beijing, 100101, P.R. China; E-mails: zhangrh@igsnrr.ac.cn; suhb@igsnrr.ac.cn; chensh@igsnrr.ac.cn; xiaj@igsnrr.ac.cn; 2 Synthesis Center of Chinese Ecosystem Research Network, Institute of Geographical Sciences and Natural Resources Research, Chinese Academy of Sciences, Beijing, 100101, P.R. China; E-mail: sunxm@igsnrr.ac.cn

**Keywords:** Two-layer model, surface evapotranspiration, surface energy balance, Bowen Radio, trapezoid method

## Abstract

In order to make the prediction of land surface heat fluxes more robust, two improvements were made to an operational two-layer model proposed previously by Zhang. These improvements are: 1) a surface energy balance method is used to determine the theoretical boundary lines (namely ‘true wet/cool edge’ and ‘true dry/warm edge’ in the trapezoid) in the scatter plot for the surface temperature *versus* the fractional vegetation cover in mixed pixels; 2) a new assumption that the slope of the *T*_m_ – *f* curves is mainly controlled by soil water content is introduced. The variables required by the improved method include near surface vapor pressure, air temperature, surface resistance, aerodynamic resistance, fractional vegetation cover, surface temperature and net radiation. The model predictions from the improved model were assessed in this study by *in situ* measurements, which show that the total latent heat flux from the soil and vegetation are in close agreement with the *in situ* measurement with an RMSE (Root Mean Square Error) ranging from 30 w/m^2^∼50 w/m^2^, which is consistent with the site scale measurement of latent heat flux. Because soil evaporation and vegetation transpiration are not measured separately from the field site, *in situ* measured CO_2_ flux is used to examine the modeled *λE*_veg_. Similar trends of seasonal variations of vegetation were found for the canopy transpiration retrievals and *in situ* CO_2_ flux measurements. The above differences are mainly caused by 1) the scale disparity between the field measurement and the MODIS observation; 2) the non-closure problem of the surface energy balance from the surface fluxes observations themselves. The improved method was successfully used to predict the component surface heat fluxes from the soil and vegetation and it provides a promising approach to study the canopy transpiration and the soil evaporation quantitatively during the rapid growing season of winter wheat in northern China.

## 1. Introduction

Evapotranspiration (*λE*, soil evaporation and vegetation transpiration) from the land surface is an important link between the surface energy balance and the hydrologic cycle. Its accurate characterization is therefore very important in the study of the terrestrial ecosystem, climate dynamics and hydrologic cycle. At present, estimate of regional evapotranspiration has been made possible by using the remote sensing observations in combination with the surface meteorological data. In the past years, several remote sensing methods were developed to simulate surface-atmosphere interactions and to retrieve the terrestrial evapotranspiration over a wide range of spatial scales [1].

By treating the soil-vegetation system as a single uniform leaf, the big-leaf model simplified the mechanism of the energy exchange between the surface and the atmosphere, and therefore the regional scale evapotranspiration simulation is made. This category of models is simple and convenient to use, but the limitation is that this big-leaf approximation in the model is not applicable to surfaces with highly spatial heterogeneity due to large differences of surface energy exchange between soil and vegetation, such as in arid or semi-arid areas. Therefore, a two-layer model is proposed and the surface available energy is partitioned between soil and vegetation to overcome the limitation of the big-leaf model. These models have an improvement over the big-leaf models when applied to sparsely vegetated surfaces [2]. Another motivation to study the two-layer model is that China exhibits a highly heterogeneous land cover due to a large population and scattered residential areas. The two-layer model is more suitable in such areas.

In the existing two-layer models, the cores of the algorithm primarily lie in two aspects: (1) accurately decomposing surface temperature of mixed pixel (*T*_m_) into soil temperature (*T*_soil_) and vegetation temperature (*T*_veg_); (2) obtaining accurate surface resistances, such as aerodynamic resistance, canopy resistance, residual resistance. In recent years, many attempts had been made to investigate the two issues. For instance, Norman and Kustas [3,4,5] used remote measurements of surface directional brightness temperature and some ancillary data to obtain soil temperature and vegetation temperature (called multi-angle method), applied Beer's law to partition net radiation of mixed pixel (*R_n_*) and employed Monin-Obukhov similarity theory to compute aerodynamic resistance (*r*_a_); Zhang *et al.* [6] presented a PCACA algorithm (PCACA, Pixel Component Arranging and Comparing Algorithm) and a layered energy-separating algorithm on the basis of triangle method and Bowen-ratio energy balance method to partition surface temperature, surface albedo (*α*_m_) and net radiation of mixed pixel, and finally to estimate soil evaporation (*λE*_soil_) and vegetation transpiration (*λE*_veg_). Because the multi-angle satellite data is not always available, multi-angle method of surface temperature decomposing is limited for applications, In contrast, PCACA algorithm is more convenient because only single angle remote sensed data are required and it can be provided from most of the satellite data. Additionally, by using the layered energy-separating algorithm the core of which is Bowen-ratio energy balance method, the uncertainties in surface energy partitioning based on the Beer's law are reduced.

PCACA algorithm and layered energy-separating algorithm utilize the scatter plot of the surface temperature against vegetation fraction cover (*T*_m_ – *f* space) to determine the soil water status, like the trapezoid method. On the basis of: (1) the assumption that the configuration of *T*_m_ – *f* space is not primarily caused by differences in atmospheric conditions and soil attributes (eg. air temperature *T*_a_, aerodynamic resistance *r*_a_, surface reflectivity *α*) but by the variations of soil water availability; (2) the fact that iso-lines of equal soil water availability are nearly straight in *T*_m_ – *f* space, which was reported in previous studies on trapezoid method [7-11], Zhang *et al.* [6] indicated that *T*_soil_ values for all pixels at an iso-line are equivalent, so are for *T*_veg_ values, which is just like the case that while measuring the same area constituted by soil and vegetation at varying view angles, *T*_soil_ and *T*_veg_ are invariable and thus *T*_m_ observations only vary with vegetation fraction cover. Under this condition, *T*_soil_ and *T*_veg_ could be obtained by calculating the slopes of these iso-lines of equal soil water availability (d*T*_m_/d*f*). Detailed descriptions about the two algorithms and the trapezoid method can be found in Zhang *et al.* [6] and in Carlson *et al.* [9, 12], respectively. In this approach, an assumption of a uniform atmospheric environment and homogeneous soil surface is required. In most cases, however, this strict requirement can't be satisfied, especially on a regional scale. In addition, the identification of the trapezoid shape of *T*_m_ – *f* space in the trapezoid method requires a flat surface and a large number of pixels over an area with a wide range of soil wetness and vegetation fraction cover, which usually cannot be satisfied within a limited study area, and therefore some subjectivity would be introduced in the determination of the trapezoid shape and it will inevitably introduce some extra errors in the*T*_soil_ and *T*_veg_ calculations, The temperature separation finally influences *λE* estimate.

In this paper, to improve the accuracy of *λE* estimate using the two-layer model presented by Zhang *et al.* [6], two modifications were made to the PCACA algorithm and layered energy-separating algorithm mentioned above: (1) to ensure that the assumption that the configuration of *T*_m_ – *f* space is mainly controlled by soil water availability is reasonable, the effects of atmospheric conditions on surface temperature would be ignored by using the averaging method; (2) to identify the shape of the trapezoid bounded by true wet/cool edge' and ‘true dry/warm edge, a general method based on surface energy balance was used. Finally, comparisons between *λE* retrievals from the original model and the improved one, and *in situ λE* measurements were done to assess the improved method.

## 2. Model Descriptions

The two-layer model used in this study was presented by Zhang *et al.* [6], and in it the PCACA algorithm and layered energy-separating algorithm are the key algorithms. In the model, land cover is simplified as a mixture of two elements, namely, vegetation and bare soil. The energy fluxes are partitioned between the soil and vegetation, and energy exchange between vegetation and bare soil is negligible. Two parameters, albedo and surface temperature, are the main different characteristics of vegetation and bare soil, and lead to different interactions between them and atmosphere. Air temperature, air humidity, wind speed and aerodynamic resistance are approximated the same for vegetation and bare soil in the same pixel due to intensively atmospheric blending effect.

### 2.1 An interpretation of T_m_ – f space

**Figure 1.**
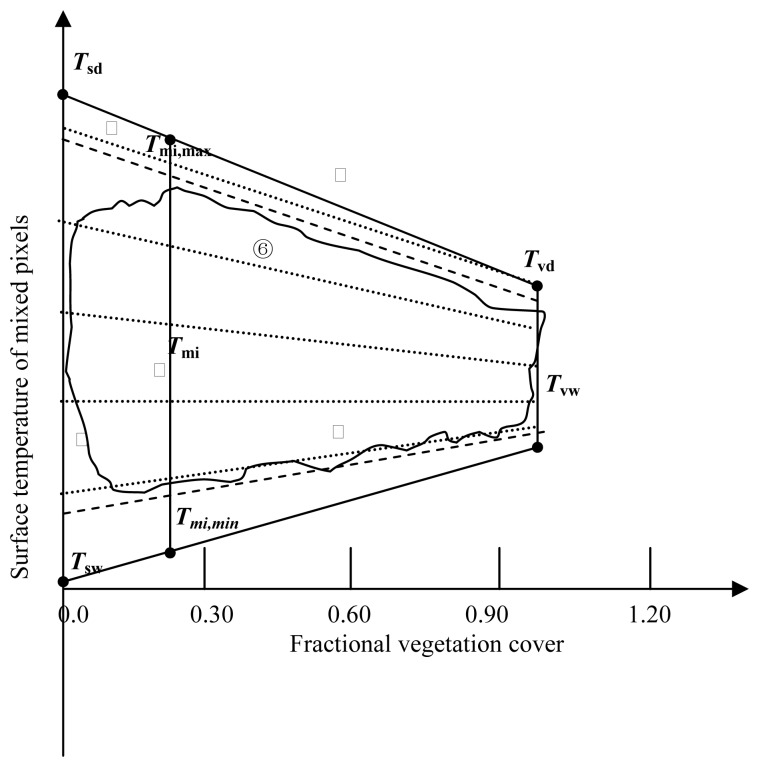
Scatter plot of surface temperature against vegetation fraction cover □ true dry edge □ observed dry edge □ observed wet edge □ true wet edge □ iso-line of equal vegetation fraction cover □ iso-line of equal soil water availability.

Figure 1 provides a conceptual illustration of *T_m_* – *f* space, where “true wet/cool edge” of the trapezoid is related to surface conditions of potential evapotranspiration and has minimum surface resistance to evapotranspiration (*r*_smin_).

Soil water content equals to field capacity; ‘true dry/warm edge’ represents zero evapotranspiration and has maximum surface resistance to evapotranspiration (*r*_smax_). If the positions of the two edges are determined, the shape and the structure of the trapezoid can be fixed and the consequent calculations of the surface heat fluxes could be done. To make the illustration easier to follow, four points are defined: *T*_sd_ and *T*_sw_ represent the points of true dry bare soil and true water saturated bare soil, respectively. *T*_vd_ represents true dry full-cover vegetation and *T*_vw_ represents true water saturated, full-cover vegetation. The above definitions all correspond to the ideal surface conditions, namely there exist driest and wettest bare soil and full-cover vegetation. In reality there are always insufficient number of pixels that can cover all kinds of soil wetness and vegetation fraction cover within the study area, which leads to a difficulty in determining “true wet/cool edge” and “true dry/warm edge”, as a result, “observed wet/cool edge” and “observed dry/warm edge” (dashed lines) are often defined according to the envelop shape of the actual scatter plot to represent actual two extreme soil moisture conditions and are used to replace “true wet/cool edge” and “true dry/warm edge” in most applications, although some errors would be introduced. Iso-line of equal vegetation fraction cover intersects “true dry edge” and “true wet edge” at true maximum temperature and true minimum temperature denoted as *T*_mi,max_ and *T*_mi,min_, respectively. It has to be noted that for the trapezoid constructed by data with coarser pixel resolution (eg. 1km), surface temperature at “true dry edge” generally is higher than that at “observed dry edge”, contrarily surface temperature at “true wet edge” is lower than that at “observed wet edge” according to the findings of Carlson [9] about the scale issues of trapezoid space. The major reason is the absence of the driest and wettest pixels due to the great mixing effect of the pixel (vegetation/bare soil).

### 2.2. PCACA Algorithm

The PCACA algorithm is a method to decompose the mixed surface temperature. As mentioned above, its physical basis is the fact that soil water status can be represented by the configuration of *T*_m_-*f* space, and like the triangle method, the crucial assumption is that surface temperature is mainly controlled by soil water availability [9, 10, 12].

The basic formulations used to decompose mixed surface temperature are shown in Eq. (1) and Eq. (2). The derivations of Eq. (2) can be found in Appendix I.

(1) $$\sigma {ɛ}_{m}T_{m}^{4}=\sigma {ɛ}_{\text{veg}}fT_{\text{veg}}^{4}+\sigma {ɛ}_{\text{soil}}\left(1-f\right)T_{\text{soil}}^{4}$$

(2) $$k=\frac{dT_{m}}{df}\approx T_{\text{veg}}-T_{\text{soil}}$$

where, *ε*_m_, *ε*_veg_ and *ε*_soil_ are the broadband emissivities of mixed pixel, vegetation and bare soil, respectively; σ is the Stefan-Boltzmann constant; d*T*_m_/d*f* represents the slope of iso-line of equal soil water availability, expressed as *k* in the remainder of the paper. In the study, constant *ε*_veg_ of 0.97 and *ε*_soil_ of 0.95 were used. By simply weighting the fractional cover for vegetation and bare soil, mixed pixel emissivity *ε*_m_ for each pixel can be computed following Eq. (3) [13].

(3) $${ɛ}_{m}=f{ɛ}_{\text{veg}}+\left(1-f\right){ɛ}_{\text{soil}}$$

From the above three equations, we can see that to solve *T*_soil_ and *T*_veg_, *k* for each pixel is the key parameter and needs to be obtained firstly.

According to Figure 1, two procedures were needed to derive *k* in the study: (1) determining the shape of the trapezoid bounded by ‘true dry edge’ and ‘true wet edge’, namely locating the ‘true dry edge’ and ‘true wet edge’ on the trapezoid, and thereby calculating the two slopes of the two bounder lines (*k*_u_ is for upper bound, *k*_L_ is for lower bound). A detailed method for this will be illustrated in section 3; (2) linearly interpolating *k* between the highest and the lowest surface temperature to obtain the slope for each pixel based on the equation below:

(4) $$k_{i}=\frac{T_{mi}-T_{mi,\min}}{T_{mi,\max}-T_{mi,\min}}\left(k_{u}-k_{L}\right)+k_{L}$$

Considering that the precise formulation of the relationship is actually unknown, linear interpolation is a reasonable approximation according to the study on the configuration of *T_m_* – *f* space by Calson [9, 10] and Moran [14].

### 2.3 Layered Energy-separating Algorithm

Essentially, the aim of the Layered Energy-separating algorithm is to calculate Bowen-ratio (*β*, the radio of sensible heat flux to latent heat flux) of soil and vegetation expressed as *β*_soil_ and *β*_veg_, respectively. By using the relationship between Water Deficit Index *(WDI*), evapotranspiration and potential evapotranspiration (*λE*_0_) illustrated by Moran *et al.* [14], Eq. (5) can be derived:

(5) $$\beta_{i}\approx \frac{T_{m,\max}-T_{m,\min}}{T_{m,\max}-T_{mi}}-1$$

where subscript *i* represents pixel *i*. From Eq.(5) we can see that *β* can be obtained conveniently from the data shown on Figure.1.

### 2.4 Estimation of Other Core Variables

After *T*_s_, *T*_v_, *β*_s_ and *β*_v_ are obtained using the above methods, net radiation at the soil surface and at the vegetation surface (*R*_n,soil_, *R*_n,veg_) can be calculated following Eqs. (6) and (7), and thereby soil heat flux and net radiation of mixed pixel can be estimated using the empirical formulation of Eq. (8) and Eq. (9), as used widely in previous studies [15-17].

(6) $$R_{n,\text{soil}}=S_{0}\left(1-\alpha_{\text{soil}}\right)+\sigma {ɛ}_{\text{sky}}T_{\text{sky}}^{4}-\sigma {ɛ}_{\text{soil}}T_{\text{soil}}^{4}$$

(7) $$R_{n,\text{veg}}=S_{0}\left(1-\alpha_{\text{veg}}\right)+\sigma {ɛ}_{\text{sky}}T_{\text{sky}}^{4}-\sigma {ɛ}_{\text{veg}}T_{\text{veg}}^{4}$$

(8) $$G\approx 0.3\left(1-0.9f\right)R_{n,\text{soil}}$$

(9) $$R_{n}=fR_{n,\text{soil}}+\left(1-f\right)R_{n,\text{veg}}$$

where *S*_0_ is the solar incident total radiation and is regarded as spatially uniform for clear sky conditions at the regional scale, usually obtained from standard meteorological station; *α*_soil_ and *α*_veg_ are the albedo of bare soil and vegetation, also calculated by the PCACA algorithm (seen from Appendix И); *ε*_sky_ is the average sky emissivity and is approximately set to 1.0 in the study. *T*_sky_ is average sky temperature and usually approximates to the temperature at 37° view sky angle [18], detailed calculation about which will be described in the Section 3.

In terms of the Bowen-ratio energy balance method, soil evaporation (*λE*_soil_) and vegetation transpiration (*λE*_veg_) can be retrieved based on Eq. (10). When energy exchange between vegetation and bare soil is neglected, *λE* of a mixed pixel can be described as a linear combination of *λE*_soil_ and *λE*_veg_, expressed as Eq. (11).

(10) $$\lambda E_{\text{soil}}=\frac{R_{n,\text{soil}}-G}{1+\beta_{\text{soil}}},\lambda E_{\text{veg}}=\frac{R_{n,\text{veg}}}{1+\beta_{\text{veg}}}$$

(11) $$\lambda E=f\lambda E_{\text{veg}}+(1-f)\lambda E_{\text{soil}}$$

## 3. Two improvements for the two-layer model

### 3.1 locating the true dry edge in T_m_ – f space

As mentioned above, the locations of true dry edge and the true wet edge are crucial in the application of PCACA algorithm. The previously used method to determine the trapezoid boundary often leads to uncertainties because of subjective judgement. To reduce the errors from this respect, a physically based method, which takes account of the surface energy balance, is presented in this study.

According to Figure.1, the four corner points, *T*_sd_, *T*_sw_, *T*_vd_ and *T*_vw_ can determine the envelop shape of the trapezoid, that is to say, as long as their values are obtained. Consequently, the true dry edge and the true wet edge can be determined. In the study, surface energy balance method was adopted to compute their values. For pixels at the true dry edge, Eq.(12) is used because *λE*=0. Substituting *R_n_*, *G*, *H* for Eq.(9), Eq.(8) and Eq.(13), we obtained Eq.(14) for *T*_sd_. In the same way, *T*_vd_ is formulated as Eq.(15).

(12) $$R_{n}-G=H$$

(13) $$H=\frac{\rho Cp\left(T_{s}-T_{a}\right)}{r_{a}}$$

(14) $$T_{sd}=\frac{0.7[S_{0}(1-\alpha_{sd})+\sigma {ɛ}_{\text{sky}}T_{\text{sky}}^{4}]+\frac{\rho Cp}{r_{\text{sda}}}T_{\text{sda}}}{\frac{\rho Cp}{r_{\text{sda}}}+0.7\sigma {ɛ}_{\text{soil}}T_{sd}^{3}}$$

(15) $$T_{vd}=\frac{0.7[S_{0}(1-\alpha_{vd})+\sigma {ɛ}_{\text{sky}}T_{\text{sky}}^{4}]+\frac{\rho Cp}{r_{\text{vda}}}T_{\text{vda}}}{\frac{\rho Cp}{r_{\text{vda}}}+0.7\sigma {ɛ}_{\text{veg}}T_{vd}^{3}}$$

where the subscripts *sd*, *vd* represent true driest bare soil and true driest full-cover vegetation, respectively. *ρ* is density of air, *c_p_* is the volumetric heat capacity of air, *r_a_* is the air dynamic resistance, *σ* is the S-B coefficient, *α*_sd_ is the albedo of dry bare soil.

From these two equations, we can see that *T*_sd_ or *T*_vd_ could not be iteratively computed until parameters of *α*_sd_, *α*_vd_, *T*_sky_, *r*_sda_, *r*_vda_, *T*_sda_, *T*_vda_ are acquired. From the *T_m_* – *f* space, the observed driest bare soil and the observed driest full-cover vegetation can be found. Although there are surface temperature differences between them and the true driest bare soil and true driest full-cover vegetation, they represent relative driest and wettest soil conditions. It means that aerodynamic resistance and air temperature of the observed driest bare soil and the observed driest full-cover vegetation are approximate to that of *T*_sd_, *T*_vd_ points under the conditions that the variations in atmospheric conditions are very small due to the spatially uniformity. In the study, we chose 50 pixels around the upper-left corner in the trapezoid representing the observed driest bare soil and 50 pixels around upper-right corner in the trapezoid representing the observed driest full-cover vegetation, and the highest *T*_a_ and *r*_a_ in each 50 pixels were selected as *T*_sda_ and *r*_sda_, *T*_vda_ and *r*_vda_, respectively. The method of retrieving the spatial distribution of *T*_a_ and *r*_a_ will be described in the following. The calculation of *r*_a_ requires *e_a_* and *r*_s_, the retrievals about which will also be described. As for *α*_sd_ and *α*_vd_, we selected the lowest albedo values of bare soil and full-cover vegetation within the whole scene of remotely sensed image (pixels) because low albedo would result in high surface temperature at the same soil water content, judging from Eq. (14) and Eq. (15). The item of $\sigma {ɛ}_{\text{sky}}T_{\text{sky}}^{4}$ represents downward long wave radiation that usually has small spatial variability for clear sky, thus can be obtained from the measurements of meteorological stations at the satellite overpass time. The following are the calculations of spatial distributions of T_a_, *e*_a_, *r*_s_ and *r*_a_:

#### a) Estimation of air temperature (*T*_a_)

Surface temperature, as a heat or cold source, influences the variations of air temperature by heating or cooling near-surface atmosphere. In most cases, high surface temperature is accompanied by high air temperature and low surface temperature is accompanied by low air temperature. By using this relationship between them and assuming the following ratio expressed in Eq. (16), an interpolation method is presented to map the distribution of air temperature. The numerator and the denominator both represent radiant emission. Because the effects of neighborhood pixels diminish as the distance to the pixel increases, a weight (*w*) derived from an inverse distance weight (IDW) method is assigned to each neighboring observations, and is given in Eq. (17).

(16) $$\frac{\sum_{i=1}^{n}w_{i}\cdot \sigma \cdot {ɛ}_{ai}\cdot T_{ai}^{4}}{\sum_{i=1}^{n}w_{i}\cdot \sigma \cdot {ɛ}_{mi}\cdot T_{mi}^{4}}=\frac{\sigma \cdot {ɛ}_{aj}\cdot T_{aj}^{4}}{\sigma \cdot {ɛ}_{mj}\cdot T_{mj}^{4}}$$

(17) $$\begin{array}{cc} w_{i}=\frac{d_{i}^{-p}}{\sum_{i=1}^{n}d_{i}^{-p}}, & {ɛ}_{a}=1.24\cdot {\left(\frac{e_{a}}{T_{a}}\right)}^{1/7} \end{array}$$

where *i* represents the pixel where air temperature measurement is made, *j* indicates the pixel where no measurements are available and estimate is required; *n* is the number of air temperature observations; *d* is the distance between pixel *i* and pixel *j*; *p* is an exponent. The higher the exponent is, the larger the influence of the closest observations on estimate is; *p* is set to 2 in the study; σ is the Stephen Boltzmann constant; *ε_a_* is the emissivity of air calculated from Eq. (17) [19].

#### b) Estimation of actual vapor pressure near surface (*e*_a_)

Like the relationship between air temperature and surface temperature, there are also strong interactions between near-surface actual vapor pressure and soil moisture. If there is no horizontal and vertical advection, vapor in the atmosphere mainly comes from soil water by an evapotranspiration process. Contrarily, the vapor gradient between surface and air influences the intensity of evapotranspiration. By assuming the following ratio relationship between near-surface actual vapor pressure and soil water, we interpolated *e*_a_, where soil moisture status was characterized quantitatively by the soil apparent thermal inertia:

(18) $$\frac{Rn}{2\left(T_{s}-T_{\min}\right)\Delta t^{\frac{1}{2}}}\cdot \frac{\sum_{i=1}^{n}w_{i}\cdot e_{ai}}{\sum_{i=1}^{n}w_{i}\frac{R_{ni}}{\left(T_{mi}-T_{\min}\right)}}=\frac{e_{aj}}{\frac{R_{nj}}{\left(T_{mj}-T_{\min}\right)}}$$

where *i*, *j*, *w*_i_, *T_s_* have the same meanings as in Eqs. (16) and (17); *T*_min_ is the minimum surface temperature during the daytime which usually occurs before sunrise when *R*_n_=0 and for all pixels it can be assumed to be the same value [20]. Δ*t* is the time interval between sunrise and the satellite overpass time.

#### c) Estimation of surface resistance to evapotranspiration (*r*_s_)

In the retrieval of evapotranspiration, *r*_s_ is used to correct the difference between the vapor pressure at the surface (*e*_s_) and the saturated vapor pressure at the evaporating front (*e*_s_*). In theory, *r*_s_ ranges from ∞ to 0 corresponding to the surface conditions of potential evapotranspiration and zero evapotranspiration, respectively, namely the true dry edge and the true wet edge. However, in reality the condition of zero evapotranspiration (r_smax_ = ∞) rarely occurs for vegetated surface even in semi-arid environment primary due to root zone soil water uptake, consequently, we selected a pixel closest to the observed driest bare soil where a meteorological station is located to calculate *r*_smax_ by *λE*, *e*_a_, *u* measurements at the satellite overpass time. *r*_smax_ is about 1000 (s m^-1^) according to the calculation, which is in agreement with Qiu's observations [21]; *r*_smin_ is set to 0 in the study.

After the upper and lower limits of *r*_s_ are determined, *r*_s_ is interpolated linearly within each *f* interval between the lowest and the highest temperature. Following the illustrations in Figure 1, *r*_si_ for a pixel at (*f*_i_ , *T*_i_) equals:

(19) $$r_{si}=\frac{T_{si}-T_{si,\min}}{T_{si,\max}-T_{si,\min}}\left(r_{s\max}-r_{s\min}\right)+r_{s\min}$$

The interpretation is similar to the method used by Stisen *et al.* [11]. The difference is that Stisen *et al.* used Priestly-Taylor parameter to represent an effective surface resistance to evapotranspiration.

#### d) Estimation of aerodynamic resistance (*r*_a_)

Besides air temperature, aerodynamic resistance is a site-specific variable and can not be retrieved directly by remote sensing. Although Monin-Obukhov similarity theory has been widely used to estimate it, the accurate calculations for the spatial distributions of roughness length (*Z*_0_), wind speed (*u*) and the atmospheric stability parameters are very difficult. In this study, we adopted the energy balance method.

An expression of *r*_a_ is obtained on the basis of the energy-balance by substituting *R*n, *G*, *H* and *λE* for Eq. (9), Eq. (8), Eq. (13) and Eq. (20):

(20) $$\lambda E=\frac{\rho c_{p}\left(e_{s}^{*}-e_{a}\right)}{\gamma \left(r_{s}+r_{a}\right)}$$

(21) $$\begin{array}{l} a_{1}r_{a}^{2}+a_{2}r_{a}+a_{3}=0\\ a_{1}=(0.7+0.27f)\cdot \gamma \cdot R_{n}\\ a_{2}=(0.7+0.27f)\gamma \cdot R_{n}\cdot r_{s}-\rho c_{p}\gamma (T_{m}-T_{a})-\rho c_{p}(e_{s}^{*}-e_{a})\\ a_{3}=-\rho c_{p}\gamma (T_{m}-T_{a})\cdot r_{s} \end{array}$$

where *γ* is the psychometric constant, *e*_s_* is the saturated vapor pressure at *T*_m_ estimated using the classic formulation with regard to surface temperature, as Eq. (22), other parameters have the same meanings as the above illustrations. Here, *T*_a_, *e*_a_ and *r*_s_ are estimated by the approaches mentioned previously.

(22) $$e_{s}^{*}=6.1078\exp(\frac{17.27\cdot T_{m}}{T_{m}+237.3})$$

Above all, all necessary parameters to calculate *T*_sd_, *T*_vd_ can be retrieved according to the Eqs. (12) – (22) in combination with limited ancillary data. Here, *T*_m_ and *α*_m_ is obtained from MODIS (Moderate Resolution Imaging Spectroradiometer) standard land data products, MOD11 and MOD02 [22, 23], through the NASA Earth Observing System Data Gateway.

### 3.2 Locating the true wet edge in T_m_ – f space

Many studies [9, 24, 25] indicated that the dry edge is more evident than the wet edge in the *T*_m_– *f* space. There often exist outlying points exhibiting a tail toward low values of temperature and *f*. Such points may represent anomalous surfaces mainly due to cloud contamination and usually are discarded from the analysis [10]. Thus, although *T*_sw_ and *T*_vw_ also can be parameterized on the basis of energy balance equation like *T*_sd_ and *T*_vd_, the inputs (*α*_sd_, *α*_vd_, *r*_sda_, *r*_vda_, *T*_sda_, *T*_vda_) cannot be obtained by the above methods. Taking account of the fact that the surface radiant temperature of dense vegetation is very close to the ambient air temperature [9, 26, 27], we took the average air temperature at *f* =1 as *T*_vw_. As for *T*_sw_, we adopted an approximation of using the surface temperature of standing water body (such lake) as the surface temperature of true wet bare soil *T*_sw_, that is to say, standing water body is regarded as the surface of potential evapotranspiration. In fact, it is not uncommon to find some patches of standing water body in a remote sensed image. In this study, the surface temperature of Dongping Lake (35.965°, 116.81°; water area: 209 km^2^) was used as *T*_sw_. In applications, we found that the pixels with mixed surface temperatures below the true wet edge all scattered around the cloud pixels and the coastline pixels.

### 3.3 Physical illustrations for the uncertainties using the above locating methods

**Figure 2.**
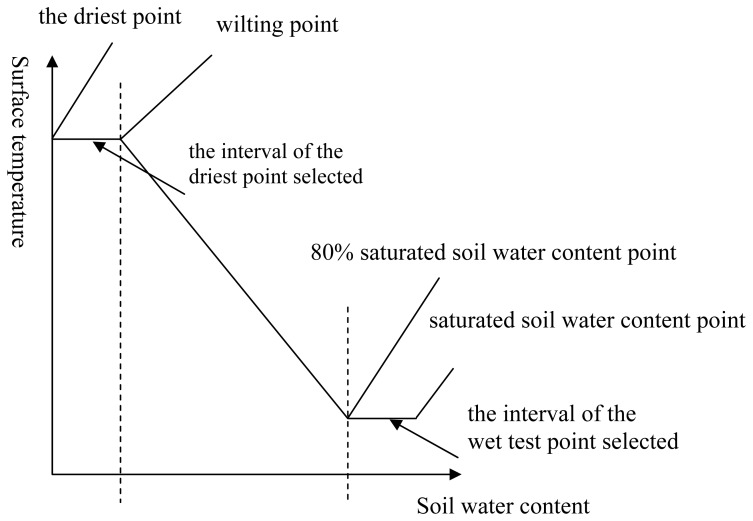
Relationship between surface temperature and soil water content.

Using the above methods, the positions of the true dry edge and the true wet edge in the triangle can be located. When surface radiant temperature is mainly dominated by surface soil water content, the following relationship between surface temperature and soil water content is tenable (Figure 2), which has been illustrated in the Qiu's experiment [21]. Figure 2 suggests that when the soil water content ranges from 80% to 100% of the soil saturation, evapotranspiration will happen approximately at the level of potential Evapotranspiration. On the contrary, when the range of the soil water content is from the wilting point to the driest, evapotranspiration is close to zero, and in the two extreme conditions, surface temperature can be regarded as a constant due to the same evaporative cooling effect. On the basis of this, although the true driest and the true wettest points can't be determined using the above methods, the errors will be acceptable in the determination of *T*_sd_, *T*_sw_, *T*_vd_ and *T*_vw_ due to the constant surface temperature.

### 3.4 Elimination of the effects of atmospheric conditions on surface evapotranspiration

Same as the triangle method, an important assumption for the PCACA algorithm is that the surface evapotranspiration is primarily constrained by soil water availability, based on which *T*_soil_ values for all pixels with an equal water availability are identical, so are for *T*_veg_ values, that is to say, the configuration of *T*_s_ and *f* is mainly caused by the variation of the soil water availability and is irrelevant to the differences in atmospheric conditions and surface properties [11]. However, in reality it is not true that the atmospheric conditions and surface properties are entirely homogeneous within the large study area. There exist some differences eg. in wind speed, air temperature, surface roughness length and surface albedo, which are all site-specific variables. As a result, it will lead to the the variability of the surface evapotranspiration and thereby influence the surface temperature and the configuration of *T_s_* and *f*. To satisfy the assumption, the effects of these factors need to be eliminated.

In the study, the effects of four controlling factors (*r*_a_, *e*_a_, *T*_a_ and albedo) on surface temperature were eliminated. According to the assumption, *r*_a_, *e*_a_, *T*_a_ and albedo values of each pixel should be equal, namely they are spatially homogeneous, to ensure that only water availability controls surface temperature. To meet this requirement, the average values of *r*_a_, *e*_a_, *T*_a_ and albedo in the image are assigned to each pixel and a new mixed surface temperature (*T*_mi_’ ) is calculated based on energy balance equation. *T*_mi_’ is the assumed temperature controlled only by soil water availability and therefore the new trapezoid constructed by *T*_m_ and *f* is more meaningful for the PCACA algorithm. It has to be noted that although the configuration of *T*_m_’ – *f* space is different from that of the *T*_m_ – *f* space, the locations of the true dry edge and the true wet edge in the trapezoid don't change since the two theoretical extreme soil water status at the satellite overpass time are the same. On the basis of *T*_m_’, *T*_veg_’ (vegetation temperature controlled only by soil water availability) and *T*_soil_’ (soil temperature controlled only by soil water availability) can be obtained. Now the problem is that the calculated *T*_veg_’ and *T*_soil_’ cannot represent the actual temperature of vegetation and soil after the above transformation., Therefore they can't be directly used in the consequent calculations. To estimate the true vegetation temperature and true soil temperature (*T*_veg_ and *T*_soil_) from *T*_veg_’ and *T*_soil_’, the following method is used.

Assuming that the thermal energy fraction assigned to the vegetation and the soil are constants, the following expression [Eq. (23)] can be approximated. The left item represents the proportion of thermal energy difference for vegetation or soil to the total thermal energy difference, and the right item represents the proportion of vegetative or soil thermal energy to the total thermal energy. It suggests that changes of environmental conditions bring same effects on vegetation temperature, soil temperature and mixed surface temperature, for example, *T*_soil_, *T*_veg_ and *T*_m_ values would all increase with the increase of solar radiation intensity and all influenced by wind speed. It is not likely to happen that the *T*_soil_ increases, but *T*_veg_ decreases in the same atmospheric conditions. Therefore, the assumption is reasonable in practice and it is different from Beer's law using fractional vegetation cover as the weight to partition thermal energy. Using this equation, *T*_soil_^4^ - *T*_veg_^4^ can be solved as Eq. (24).

(23) $$\begin{array}{cc} \frac{T_{\text{veg}}^{4}-T_{\text{veg}}^{\prime 4}}{T_{m}^{4}-T_{m}^{\prime 4}}\approx \frac{T_{\text{veg}}^{4}}{T_{m}^{4}}, & \frac{T_{\text{soil}}^{4}-T_{\text{soil}}^{\prime 4}}{T_{m}^{4}-T_{m}^{\prime 4}}\approx \frac{T_{\text{soil}}^{4}}{T_{m}^{4}} \end{array}$$

(24) $$T_{\text{veg}}^{4}-T_{\text{soil}}^{4}=\frac{T_{m}^{4}}{T_{m}^{\prime 4}}(T_{\text{veg}}^{\prime 4}-T_{\text{soil}}^{\prime 4})$$

Combined with Eqs. (1) and (24), *T*_soil_ and *T*_veg_ can be solved. *T*_soil_ and *T*_veg_ can be directly obtained from Eq. (23), at the same time the uncertainties of the solution can be reduced to the minimum by this method because the difference between *T*_soil_ and *T*_veg_ is used instead of the absolute temperatures and Eq.(1) helps to maintain the thermal energy balance in the calculation of *T_s_*_oil_ and *T*_veg_ values.

## 4. Study area and field measurements

The study area is located in the North China Plain and ranges from 35.2N to 40.84N in latitude, from 113.68E to 119.54E in longitude. The land use in the area is dominated by the rotating cropping of winter wheat and summer maize. Millet, soybean and cotton are also scattered planted in summer [28]. According to the traditional tillage practice, winter wheat is sown in early October, harvested in early or mid June next year, and summer maize is planted in early to mid June and harvested at the end of September. The soil is mostly silt, light loam and medium loam. Annual precipitation is about 600 mm, more than 50% of which falls during the summer monsoon between July and September. The groundwater table varies from 1.5 m to 3.5 m with an average of 2.5 m.

**Figure 3.**
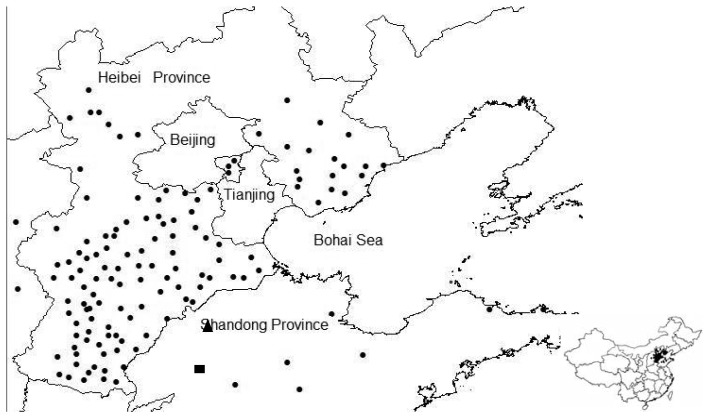
Spatial distribution of standard meteorological stations in the study area.

In the study, field measurements from 135 standard meteorological stations were used. The measurements include air temperature and actual vapor pressure at 2 m height above the surface, solar incoming radiation, surface radiative temperature, wind speed, upward longwave radiation, upward shortwave radiation, downward longwave radiation and downward shortwave radiation. Figure.3 shows the spatial distribution of these stations in the study area, where the triangle symbol (▲) marks the location of Yucheng Agro-ecosystem Station that can represents the largest agricultural area in the North China Plain [29]. Sensible heat flux and latent heat flux have been continuously measured by the Eddy Correlation (EC) system which is composed of a 3D sonic anemometer and an open path CO_2_/H_2_O analyzer since 2002. *H*, *λE* and CO_2_ fluxes were originally sampled at 10 Hz and values averaged over 30 min were used in the study to validate the above mentioned methods. The rectangle symbol (■) shows the location of Dongping Lake. Water surface temperature has been measured for seven years since 2001 and was used to validate the MODIS land surface temperature products used in this study, and thereby to ensure the accuracy of *T*_sw_ values.

## 5. Satellite data

MODIS land data products, including MOD11 (Land Surface Temperature), MOD03 (Geolocation Data Set), MOD05 (Total Precipitable Water), MOD02 (Calibrated Geolocated Radiance) and MOD35 (Cloud Mask), for clear sky during springtime between Mar and June when winter wheat is the dominant crop were used in the study. The visible channels of MOD02 were processed with atmospheric correction using the SMAC algorithm [30] with the combination of the MOD03 and MOD05 data. The cloud was masked out based on MOD35 data. The basic variables used as inputs in the model consist of albedo, fractional vegetation cover and surface temperature, based on which the calculation of *R*_n_, PCACA algorithm and Layered Energy-separating Algorithm were applied.

The instantaneous surface albedo was obtained by averaging reflectance values for several visible and near-infrared channels with the wavelength as the weight. This would introduce some errors because channel reflectance values observed by a satellite are reflectance at only one Sun-target-sensor configurations and it is generally different from the hemispherical reflectance. But we assumed its influence to be small as pointed by Nishida [27]. The fractional vegetation cover was estimated from the normalized difference vegetation index (*NDVI*) [27], as Eq.(25).

(25) $$f=\frac{\text{NDVI}-{\text{NDVI}}_{\min}}{{\text{NDVI}}_{\max}-{\text{NDVI}}_{\min}}$$

where *NDVI*_max_ and *NDVI*_min_ are *NDVI* values for full cover vegetation (*f*=1) and bare soil (*f*=0). According to the field *NDVI* measurements performed at Yucheng station, *NDVI*_min_=0.09 and *NDVI*_max_=0.78. Other variables, such as *R*_n_, *G*, *T*_a_, *e*_a_, *r*_s_, were obtained using the above mentioned methods.

## 6. Results and Discussion

**Figure 4.**
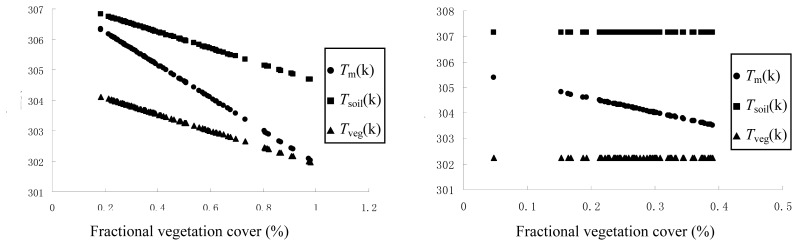
Comparisons between *T*_soil_, *T*_veg_ obtained from the original model and *T*_soil_’, *T*_veg_’ obtained from the improved model at iso-line of equal water availability.

Figure 4 shows the comparisons between *T*_soil_, *T*_veg_ obtained from the original model and *T*_soil_’, *T*_veg_’ obtained from the improved model at an iso-line of equal water availability.

Apparently, the results of *T*_soil_’, *T*_veg_’ are in closer agreement with the above assumptions that soil surface temperature for all pixels at an iso-line are identical, so are the vegetative surface temperature. It provides a better physical foundation for the model.

**Figure 5.**
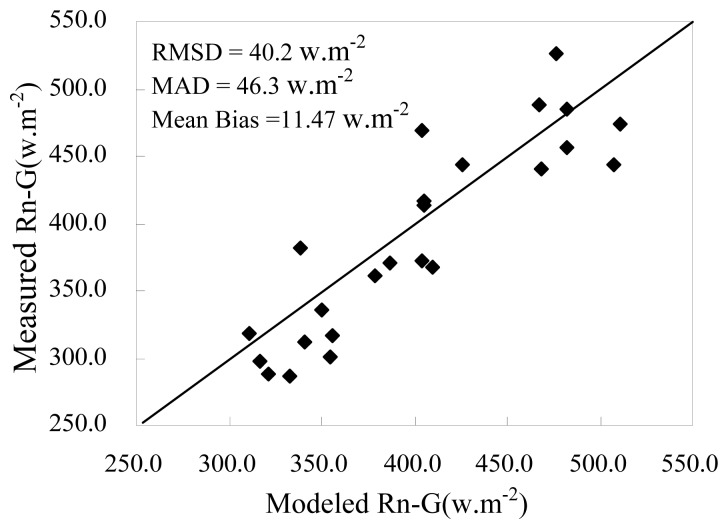
Modeled versus measured available energy, *R*_n_-*G*. The line represents a 1: 1 relationship.

**Figure 6.**
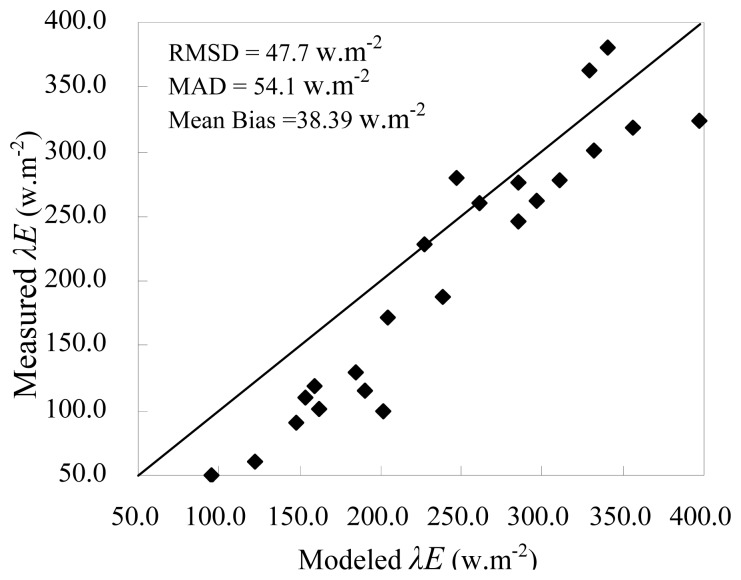
Modeled versus measured latent heat flux, *λE*. The line represents a 1: 1 relationship.

Due to the absence separate measurement of the soil evaporation, soil heat flux, vegetation transpiration and vegetation heat flux from the field sit, estimates of *R*_n_-*G* and *λE* from the model were compared to the measurements from Yucheng station, as shown in Figure 5 for available energy (*R*_n_-*G*), in Figure 6 for *λE*.

**Figure 7.**
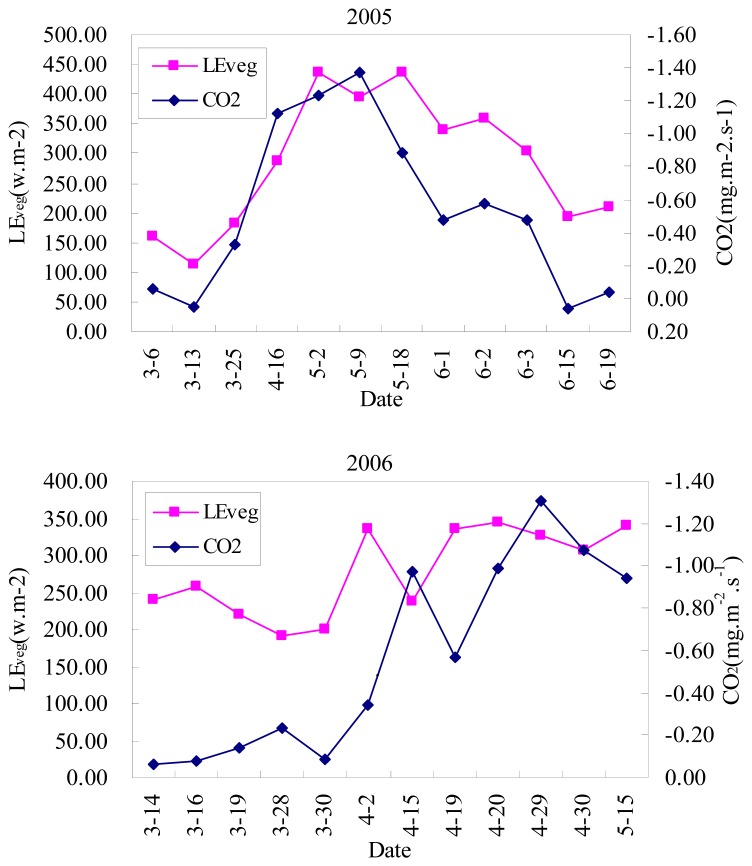
Seasonal variation of the modeled *λE*_veg_ and measured CO_2_ flux during winter wheat growing period 2005 and 2006 (from March to June).

Because direct comparison of *λE*_soil_ and *λE*_veg_ cannot be performed, the relationship between modeled *λE*_veg_ and measured CO_2_ were used to indirectly validate the two-layer model since CO_2_ fluxes are closely related to *λE*_veg_[31, 32]. Figure 7 shows the scatter plot between modeled *λE*_veg_ and measured CO_2_ flux. Note that the minus sign (-) for CO_2_ fluxes means that the flux transferring direction is from up to down. The performance of the model was evaluated using the root mean squares difference (RMSD) and the mean absolute difference (MAD), which are defined as Eq.(26) and Eq.(27), respectively.

(26) $$\text{RMSD}={\left[\sum_{i=1}^{n}{\left(P_{i}-O_{i}\right)}^{2}/n\right]}^{1/2}$$

(27) $$\text{MAD}=\sum_{i=1}^{n}\left|P_{i}-O_{i}\right|/n]$$

where *n* is the number of observations, *P* represents the model estimated value, *O* represents the observed value.

The figures demonstrate that (1) no significant bias is found between the modeled and measured *R*_n_-*G* with RMSD=46.3 and MAD=40.2. The high correlation coefficient (r^2^=0.79) suggests that *R*_n, soil_ and *R*_n, veg_ calculated from *T*_soil_ and *T*_veg_ are reasonable because *R*n is calculated by them; (2) estimates of *λE* tends to overestimate for lower *λE* values and shows larger bias than *R*_n_-*G* resulting in 54.1 of RMSD and 47.7 of MAD, which implies a larger uncertainty of the model in low *λE* conditions; (3) in general, seasonal variations in modeled *λE*_veg_ and measured CO_2_ flux shows good agreement though few points in 2006 showed different variations perhaps influenced by horizontal/vertical advection or other factors. Vegetation transpiration generally increases with the crop growth, at the same time, since vegetation absorbs more CO_2_ due to the more active photosynthesis in the daytime, CO_2_ fluxes also increases. On the contrary, *λE*_veg_ and CO_2_ fluxes both became very small after crop harvest or in winter. In the study, winter wheat was harvest in June, therefore *λE*_veg_ and CO_2_ fluxes rapidly decreased on June 15 and June 19 in 2005. Figure 7 indirectly proves the soundness of the two-layer model to estimate the soil evaporation and vegetation transpiration separately.

In fact, there are two other factors which contribute to the uncertainty in the above validation. One is that point measurements usually can not represent the whole MODIS pixel (1 km^2^) because of the large scale disparity between them. The other is that the observed *R*_n_, *λE*, *H* and *G* values at field cannot meet the surface energy balance closure resulting from many possibilities, such as instrumental errors, horizontal/vertical advections [33-35], however, the basis of the model used in the study is the surface energy balance, as most remote sensing models of evapotranspiration, such as SEBS [36], SEBAL [37], N95 [3]. Therefore, it is not uncommon to see the differences between modeled fluxes and observed ones. Compared with other studies, the RMSD of 54.1 (w/m^2^) for modeled*λE* are in a acceptable range [17, 27, 38, 39].

**Figure 8.**
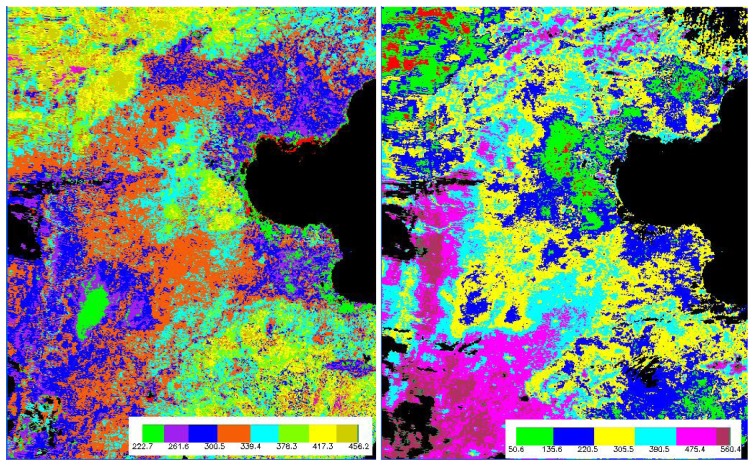
*λ*E_veg_ maps on May 2, 2005 in North China Plain retrieved by the improved model (left image) and the original model (right image).

Figure 8 shows the *λE*_veg_ maps on May 2, 2005 for the North China Plain retrieved by the improved model and the original model, respectively. Differences were found between them. The *λE*_veg_ value from the improved model varies from 222 (w/m^2^) to 456 (w/m^2^), while the original *λE*_veg_ values varied from 50 (w/m^2^) to 560 (w/m^2^). In terms of the experiences and the knowledge on the vegetation transpiration, a small *λE*_veg_ dynamical range is more reasonable in the rapid growing season of winter wheat, that is to say, the results from the improved model is better than that of the original model although it is difficult to quantitatively evaluate the two results due to the absence of field measurements of the vegetation transpiration and soil evaporation at the satellite pixel scale. Furthermore, according to the above illustrations, the physical basis of the improved model is strengthened against the original model.

## 7. Conclusions

This paper presented two improvements of a two-layer model for the estimation of the land surface heat fluxes. The weakness in the original model was identified: (1) a subjective method to determine the true boundary lines from the scatter plot for the surface temperature of mixed pixel versus the fractional vegetation cover; (2) the assumption that the configuration of *T*_m_ – *f* space is mainly controlled by soil water availability, are not physically realistic. To investigate the two issues and obtain more realistic separation of the surface temperature for the soil and vegetation components, and thereby more accurate partitioning of the surface fluxes for the soil and vegetation components, introducing the surface energy balance method solves the problem (1) and the method of eliminating the effects of four controlling factors (*r*_a_, *e*_a_, *T*_a_ and albedo) on surface temperature solves the problem (2). At the same time, the interpolation methods of *T*_a_ and *e*_a_, the methods for acquiring spatial distributions of *r*_a_ and *r*_s_ were also investigated. Finally, the improved model was applied to the North China Plain. The results showed good agreement with *in situ* terrestrial surface fluxes measurements. Furthermore, by comparing the seasonal variations of vegetation transpiration and CO_2_ flux, and the vegetation transpirations retrieved by the improved model and the original model, the effectiveness of the improved two-layer model for estimating soil evaporation and vegetation transpiration were indirectly proved.

## Appendix I

The relationship between the temperature of mixed pixel and vegetative fractional cover for 8∼14*μm* can be expressed as:

(AI.1) $$\sigma {ɛ}_{m}T_{m}^{4}=\sigma {ɛ}_{\text{veg}}fT_{\text{veg}}^{4}+\sigma {ɛ}_{\text{soil}}\left(1-f\right)T_{\text{soil}}^{4}$$

where *T*_m_, *T*_veg_ and *T*_soil_ are respectively the surface temperature of mixed pixel, vegetation canopy and soil surface. *ε*_m_, *ε*_veg_ and *ε*_soil_ are the emissivities of mixed pixel, vegetation and bare soil; σ is the Stefan-Boltzmann constant. *f* is fractional vegetation cover of mixed pixel. Evidently, *T*_veg_ and *T*_soil_ cannot be retrieved only using this equation. Computing the differential coefficient of *T*_m_ to *f* and assuming

$$\frac{dT_{\text{veg}}}{df}\approx 0,\frac{dT_{\text{soil}}}{df}\approx 0,$$

we can get Eq. (AI.2):

(AI.2) $$4{ɛ}_{m}T_{m}^{3}=\frac{dT_{m}}{df}+T_{m}^{4}\frac{d{ɛ}_{m}}{df}={ɛ}_{\text{veg}}T_{\text{veg}}^{4}-{ɛ}_{\text{soil}}T_{\text{soil}}^{4}$$

Multiplying *f* in the two sides of Eq (AI.2) and rearranging this equation, we obtained:

(AI.3) $$f(4{ɛ}_{m}T_{m}^{3}=\frac{dT_{m}}{df}+T_{m}^{4}\frac{d{ɛ}_{m}}{df})=f{ɛ}_{\text{veg}}T_{\text{veg}}^{4}-f{ɛ}_{\text{soil}}T_{\text{soil}}^{4}$$

Integrating Eq. (AI.2) with Eq. (AI.3), *T*_soil_ can be expressed as:

(AI.4) $$T_{\text{soil}}={\left\{\frac{1}{{ɛ}_{\text{soil}}}[{ɛ}_{m}T_{m}^{4}-f(T_{m}^{4}\frac{d{ɛ}_{m}}{df}+4{ɛ}_{m}T_{m}^{3}\frac{dT_{m}}{df})]\right\}}^{1/4}$$

in the same way, *T*_veg_ can be expressed as:

(AI.5) $$T_{\text{veg}}={\left\{\frac{1}{{ɛ}_{\text{veg}}}[{ɛ}_{m}T_{m}^{4}+(1-f)(T_{m}^{4}\frac{d{ɛ}_{m}}{df}+4{ɛ}_{m}T_{m}^{3}\frac{dT_{m}}{df})]\right\}}^{1/4}$$

According to Eq. (3), $\frac{d{ɛ}_{m}}{df}={ɛ}_{\text{veg}}-{ɛ}_{\text{soil}}$. In the study, $\frac{d{ɛ}_{m}}{df}=0.02$ due to the fixed values of *ε*_veg_=0.97 and *ε*_soil_=0.95. In terms of the numerical simulations, the small $\frac{d{ɛ}_{m}}{df}$ value has small influence on the difference between *T*_soil_ and *T*_veg_, therefore we ignored the difference between *ε*_veg_ and *ε*_soil_. This simplification means that Eq. (AI.1) was simplified as Eq. (AI.6), which is also reasonable in applications [5].

(AI.6) $$T_{m}^{4}=fT_{\text{veg}}^{4}+(1-f)T_{\text{soil}}^{4}$$

Furthermore, using Eq.(AI.5) minus Eq.(AI.4), the following expression can be simplified as:

(AI.7) $$T_{\text{veg}}^{4}-T_{\text{soil}}^{4}\approx 4T_{m}^{3}\frac{dT_{m}}{df}$$

In fact, $T_{\text{veg}}^{4}-T_{\text{soil}}^{4}\approx 4\left(T_{\text{veg}}-T_{\text{soil}}\right)T_{m}^{3}$ when no large temperature difference between vegetation surface and soil surface occurs, so $\frac{dT_{m}}{df}$ can be formulated as:

(AI.8) $$\frac{dT_{m}}{df}\approx T_{\text{veg}}-T_{\text{soil}}$$

## Appendix И

Albedo of mixed pixel is the weighed-average value of soil albedo and vegetation albedo, showed as Eq. (AИ.1). Computing the differential coefficient of *α*_m_ to *f* and assuming $\frac{d\alpha_{\text{veg}}}{df}\approx 0$, $\frac{d\alpha_{\text{soil}}}{df}\approx 0$, we obtained Eq.(AИ.2).

(AИ1) $$\alpha_{m}=\alpha_{\text{soil}}+f\alpha_{\text{veg}}(\text{A}H.1)$$

(AИ2) $$\frac{d\alpha_{m}}{df}\approx \alpha_{\text{veg}}-\alpha_{\text{soil}}$$

Integrating Eq. (AИ.1) with Eq. (AИ.2), *α*_soil_ and *α*_veg_ can be expressed as:

(AИ.3) $$\alpha_{\text{soil}}=\alpha_{m}-f\frac{d\alpha_{m}}{df}$$

(AИ.4) $$\alpha_{\text{veg}}=\alpha_{m}+(1-f)\frac{d\alpha_{m}}{df}$$

Evidently, as the method of decomposing mixed surface temperature *T*_m_, PCACA also can be applied to separate albedo of mixed pixel using the configuration of *α*_m_- *f* space.
